# Synthesis and Physicochemical Evaluation of Entecavir-Fatty Acid Conjugates in Reducing Food Effect on Intestinal Absorption

**DOI:** 10.3390/molecules23040731

**Published:** 2018-03-22

**Authors:** Hyuck Jun Jung, Myoung Jin Ho, Sungwan Ahn, Young Taek Han, Myung Joo Kang

**Affiliations:** College of Pharmacy, Dankook University, 119 Dandae-ro, Dongnam-gu, Cheonan, Chungnam 330-714, Korea; hyuckjun3000@naver.com (H.J.J.); butable@gmail.com (M.J.H.); tjddhks7640@naver.com (S.A.)

**Keywords:** entecavir, lipidic prodrug, lipid conjugation, oral absorption, food effect

## Abstract

The oral bioavailability of entecavir (EV), an anti-viral agent commonly prescribed to treat hepatitis B infections, is drastically reduced under a post-prandial state. This is primarily due to its low permeability in the gastrointestinal tract. To reduce the food effect on the intestinal absorption of the nucleotide analogue, four lipidic prodrugs were synthesized via the esterification of the primary alcohol of EV with fatty acids (hexanoic acid, octanoic acid, decanoic acid, and dodecanoic acid). EV-3-dodecanoate (or EV-C12) exhibited high solubility in a fed state simulated intestinal fluid (78.8 μg/mL), with the acceptable calculated log*P* value (3.62) and the lowest hydrolysis rate (22.5% for 12 h in simulated gastric fluid, pH 1.2). Therefore, it was chosen as a candidate to improve intestinal absorption of EV, especially under a fed state condition. Physical characterization using scanning electron microscopy, a differential scanning calorimeter, and X-ray powder diffraction revealed that EV-C12 had a rectangular-shaped crystalline form, with a melting point of about 170 °C. In a release test in biorelevant media, such as fasted and fed state-simulated intestinal and/or gastric fluid, more than 90% of the prodrug was released within 2 h in all media tested. These data suggest that this lipidic prodrug might have the potential to alleviate the negative food effect on the intestinal absorption of EV with increased therapeutic efficacy and patient compliance.

## 1. Introduction

Entecavir (2-amino-9-[(1*S*,3*R*,4*S*)-4-hydroxy-3-(hydroxymethyl)-2-methylidenecyclopentyl]-3*H*-purin-6-one, EV) has been frequently prescribed to treat infections caused by the hepatitis B virus (HBV) [1,2]. This oral nucleotide analog can selectively hinder HBV by restraining three steps of viral replication procedures: Base priming, reverse transcription of the negative strand, and synthesis of the positive strand of HBV DNA [3]. In several clinical studies, oral EV therapy (marketed under trade name of Baraclude^®^, Bristol–Myers Squibb, NY, USA) can markedly restore liver histology after 48 weeks of therapy, compared to lamivudine therapy. Moreover, the proportion of patients with undetectable HBV DNA, determined by both DNA signal amplification assay and polymerase chain assay, are drastically higher than those treated with lamivudine for 48 weeks after daily intake [4,5,6].

However, one of the hindrances in EV oral therapy is the food effect on intestinal absorption, in which oral absorption of this biopharmaceutics classification system (BCS) ΙIΙ compound is marked by reduced food consumption, especially after a high-fat meal. The anti-viral agent has a hydrophilic weak base with high solubility (2.4 mg/mL in distilled water at 25 °C, pKa value of 10.5) and a low log*P* value of −1.110 (pH 4 to 10) [7]. Oral EV administration at a dose of 0.5 mg with a high-fat meal (945 kcal, 54.6 g fat) experiences a delay in absorption (1.0–1.5 h fed vs. 0.75 h fasted), a decrease in maximum drug concentration in plasma (44–46%), and a decrease in the extent of drug absorption (18–20%) [8]. This negative food effect on the intestinal absorption of EV is probably due to the poor permeability of this drug in combination with post-prandial changes (pH, motility, transient times, and viscosity) in the gastrointestinal tract [9,10,11]. To lessen the negative food effect on oral EV therapy, it is indicated that it should be taken on an empty stomach, at least 2 h after a meal, and/or at least 2 h prior to the next meal [12]. However, this dosing regimen impairs patient compliance, which ultimately reduces the overall therapeutic efficacy. Moreover, in cases of oral EV therapy, reduced intestinal absorption with food resulted in lower efficacy in lamivudine-refractory patients [8]. Thus, food restrictions in EV therapy have led to the need for an alternative EV dosage form, minimizing negative food effects on intestinal absorption.

Several approaches have been explored to improve intestinal absorption of hydrophilic compounds with low permeability, such as co-administration with permeation enhancers, ion-pairing, prodrugs, and nanocarrier-based approaches [13,14,15]. Lipidic prodrugs are chemical entities with two distinct parts: A drug covalently bound to a lipid moiety, i.e., a fatty acid (FA), and a glyceride or a phospholipid. These prodrugs have been designed to improve intestinal absorption of poorly permeable compounds [16,17]. The modification of hydrophilic compounds with FA can increase their lipophilicity, thereby increasing the membrane permeability of parent compounds with poor oral absorption. After intestinal absorption, inactive lipidic moiety is cleaved from a parent compound by spontaneous enzymatic transformation and/or hydrolysis in biological fluids, resulting in the release of a free therapeutic agent. Xiao et al. reported significant intestinal absorption enhancement of chondroitin sulfate, a hydrophilic compound, by chemical modification with FA, following intragastric administration [18]. Moreover, the oral bioavailability of adefovir dipivoxil was significantly higher (59%) than that of the hydrophilic parent compound (12%) in humans [19].

Based on these findings, this study hypothesized that the modification of the oral nucleotide analog EV with FA might improve its lipophilicity, thereby leading to a positive effect on its intestinal absorption, especially under a fed state. Thus, the objective of this study was to synthesize EV-FA conjugates via esterification of the primary alcohol of EV with diverse fatty acids (hexanoic acid, octanoic acid, decanoic acid, and dodecanoic acid). These conjugates were named EV-C6, EV-C8, EV-C10, and EV-C12. Their physicochemical properties were comparatively evaluated in terms of aqueous solubility in biorelevant media, partition coefficient (log*P*), and the degree of hydrolysis in simulated gastric fluid. Moreover, the physical characteristics and in vitro dissolution profiles of an optimized lipidic prodrug under various conditions were further investigated.

## 2. Results and Discussion

### 2.1. Chemistry

**Figure 1:**
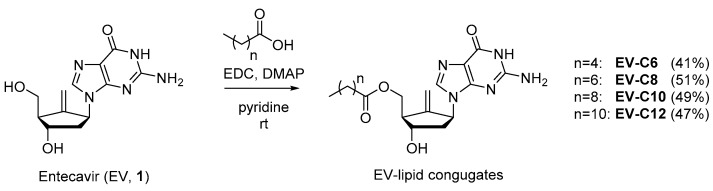
Schematic of synthesis of entecavir-fatty acid (EV-FA) conjugates.

Synthesis of EV-FA conjugates was conducted using C6 to C12 FAs, as shown in Figure 1. It was expected that the primary alcohol of EV would react with FA more preferentially than with secondary alcohol and amines of guanine moiety. In line with our expectations, mono-esterified compounds in moderate yields were obtained when EV reacted with an equivalent amount of FA in pyridine along with two equivalent amounts of EDC (1-ethyl-3-(3-dimethylaminopropyl)carbodiimide) and a catalytic amount of DMAP (*N*,*N*-dimethylaminopyridine). The structures of these synthetic compounds were confirmed by a careful NMR spectral analysis made in comparison to the spectral data of EV.

### 2.2. Determination of Aqueous Solubility, logP, and the Acidic Hydrolysis of EV-FAs

Table 1 shows the apparent solubility of EV-FA conjugates in various biorelevant media at 37 °C. In media simulating a fasting state (fasted state simulated gastric fluid and fasted state simulated intestinal fluid, or FaSSGF and FaSSIF), EV derivatives showed low water solubility, ranging from 3 to 10 μg/mL. Considering the high aqueous solubility of EV (above 1600 μg/mL in all media tested, Table 1), FA modification drastically decreased water solubility by increasing hydrophobicity. On the other hand, the aqueous solubilities of EV-FAs in fed state simulated gastric fluid (FeSSGF) and fed state simulated intestinal fluid (FeSSIF) mimicking fed conditions were much higher (24.9~78.8 μg/mL) than those in FaSSGF or FaSSIF. The apparent solubility in FeSSGF or FeSSIF was found to be linearly increased with the increase in the chain length of the hydrocarbon. An increase in the chain length of FA proportionally increased the lipophilicity of these compounds, thus facilitating incorporation of these lipidic conjugates into the mixed micellar structure formed by sodium taurocholate and deoxycholate. It is well known that lecithin and sodium taurocholate improve the wettability of hydrophobic compounds in aqueous media and enhance their solubility by locating the compounds into mixed micelles [20].

Log*P* is commonly used to predict and/or understand the intestinal absorption behavior of drug molecules, as the lipophilicity of a compound is a major factor in determining absorption [21]. According to Lipinski’s Rule of 5, the log*P* of a compound intended for oral administration should be below 5 [22]. To predict the lipophilicity of compounds, Clog*P*, one of common log*P* prediction algorithms was used [23]. All Clog*P* values for the four synthesized chemicals were below 5, in the following order: EV-C12 > EV-C10 > EV-C8 > EV-C6. Clog*P* values escalated with an increase in the chain length of FA. EV-C12 showed the highest Clog*P*, consistent with its lowest solubility in FaSSGF and highest solubility in FeSSGF and FeSSIF. On the other hand, EV-C6 showed the lowest Clog*P* value (0.45), consistent with its lowest solubility in fed state simulating media.

**Figure 2:**
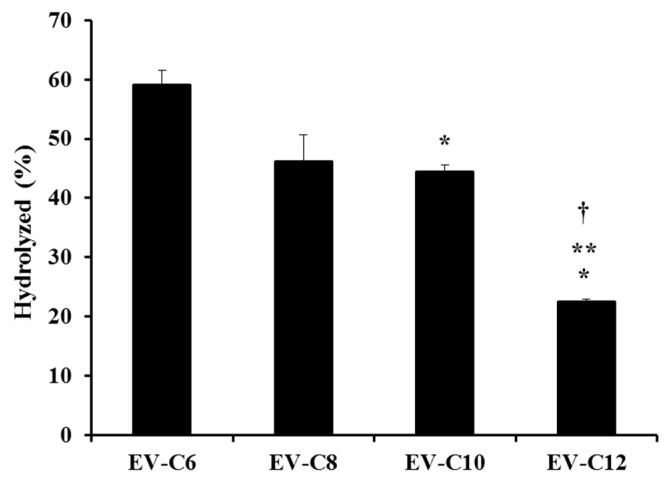
Percentage of FA conjugates hydrolyzed in simulated gastric fluid after 12 h. * *p* < 0.05 compared to EV-C6, ** compared to EV-C8, and † compared to EV-10.

Esters are labile bonds that are prone to chemical and enzymatic hydrolysis [24]. Generally, a prodrug is preferably absorbed in an intact form in the stomach and/or small intestine. After intestinal absorption, inactive moiety is cleaved from the active parent compound by enzymatic transformation and/or hydrolysis in blood, resulting in the release of a free therapeutic agent after oral absorption [25]. The degree of hydrolysis (%) of lipidic prodrugs in simulated gastric fluid (pH 1.2) is shown in Figure 2. Intact prodrug concentrations in acidic media after 12 h of incubation were normalized by the initial starting concentration (in terms of percentage). The degree of hydrolysis was gradually decreased as the length of the carbon chain was increased. The hydrolyzed percentages of EV-C6, EV-C8, EV-C10, and EV-C12 were 59.1, 46.2, 44.4, and 22.5%, respectively. This tendency is quite consistent with previous reports showing that acetate, butyrate, caprylate, and dodecanoate esters of a coumarin dye are hydrolyzed within a pH range of 8.8 to 9.8, with hydrolysis rate being the highest for acetate but the lowest for dodecanoate [26]. From these findings, with EV-C12 exhibiting high solubility under a fed state (78.8 μg/mL in FeSSIF) with an acceptable Clog*P* value (3.62), and the lowest hydrolysis rate (22.5% for 12 h in simulated gastric fluid, pH 1.2), it was selected as a prodrug candidate to improve intestinal absorption of EV, especially under a fed state.

### 2.3. Physicochemical Characteristics of EV-C12

**Figure 3:**
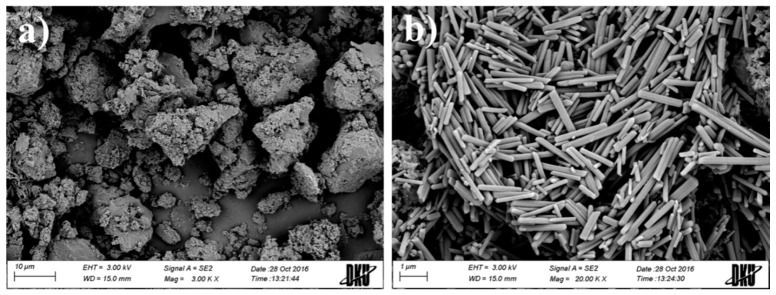
Representative SEM images of EV-C12 powder with (**a**) 3000× and (**b**) 20,000× magnification.

The physicochemical characteristics of EV-C12 were evaluated in terms of morphology, thermal properties, and X-ray diffraction patterns. The appearance of this lipidic prodrug in a solid state was characterized by SEM. This prodrug powder was mainly 2–20 μm in size, showing a rough and irregular surface (Figure 3a). In a magnified image, it was observed that uniform and rectangular-shaped crystals gathered to form the drug powder (Figure 3b). The diameter of these rectangular shaped crystals was about 1–5 μm. Their thickness was less than 0.3 μm. The rod shape of the EV-C12 crystal was analogous to that of other FA conjugates, such as pulverized riboflavin laurate [27]. The distinctive crystalline shape of this drug powder suggests that EV-C12 exists in a crystalline state.

**Figure 4:**
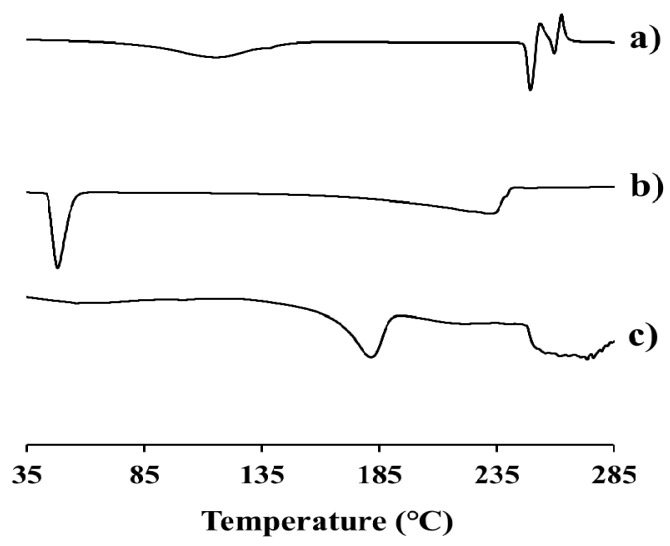
DSC thermograms of (**a**) EV powder, (**b**) dodecanoic acid, and (**c**) EV-C12 powder.

Differential scanning calorimetry (DSC) curves of EV, intact dodecanoate, and EV-C12 powders at temperature of 35 to 285 °C are shown in Figure 4. The sharp endothermic peak of the parent drug appeared at 247 °C (Figure 4a). This is quite analogous to the value previously reported (244 °C) [28]. On the other hand, modification with dodecanoate, a FA with a melting point of 43 °C (Figure 4b), lowered the melting point of this lipidic prodrug to 170~180 °C (Figure 4c). This result is consistent with an earlier study showing that the melting point of paliperidone (168~173 °C) declines to 110~115 °C after the conjugation of palmitic acid via ester linkage [29].

**Figure 5:**
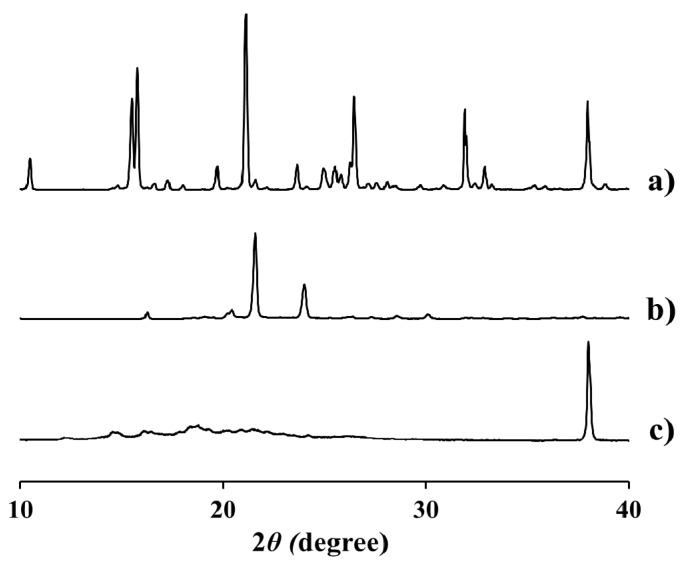
Diffraction pattern of (**a**) EV powder, (**b**) dodecanoic acid, and (**c**) EV-C12 powder.

The X-ray diffraction (XRD) patterns of EV, intact dodecanoate, and EV-C12 powders are shown in Figure 5. XRD analysis provides evidence of the structure, preferred crystal orientations, average grain size, and crystal defects of materials [30]. EV powder had a highly-crystalline state. It retained unique diffraction peaks at 2θ equal to 15.8°, 21.1°, 26.5°, 32.0°, and 38.0° (Figure 5a). Dodecanoate showed distinctive peaks at 21.6° and 24.1° (Figure 5b). On the other hand, a sharp peak was only observed at 38.0° for EV-C12 powder (Figure 5c). The broad endothermic peak over the range from 170 to 180 °C and the simple diffraction pattern suggest that this prodrug is in crystalline form, but the degree of crystallinity is lower than its parent compound.

### 2.4. In Vitro Dissolution Profile in Biorelevant Media

**Figure 6:**
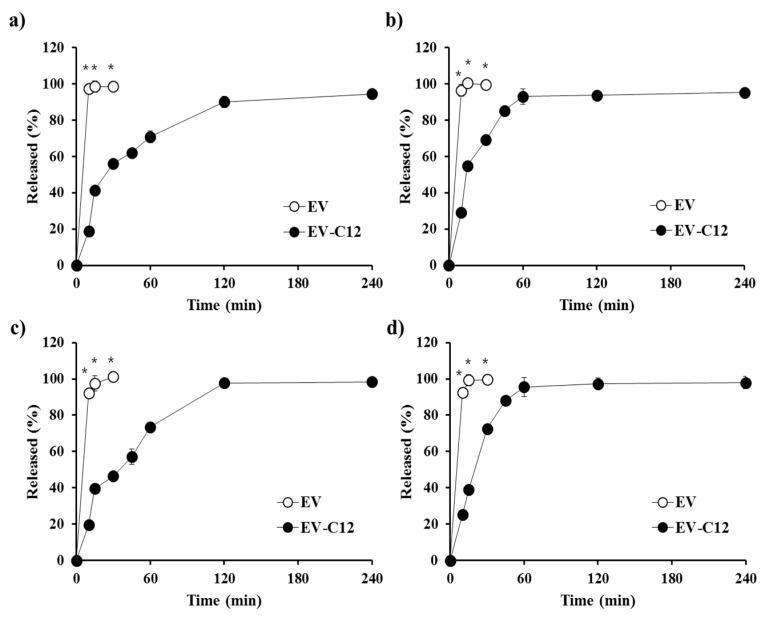
Dissolution profiles of EV and EV-C12 in different biorelevant dissolution media such as (**a**) FaSSGF, (**b**) FeSSGF (**c**) FaSSIF, and (**d**) FeSSIF. Data are expressed as mean ± S.D. (*n* = 3). * *p* < 0.05, compared to EV-12.

The dissolution profile of the parent compound EV and the lipidic prodrug EV-C12 was comparatively assessed in various biorelevant media such as FaSSGF, FaSSIF, FeSSGF, and FeSSIF. EV was released completely within 15 min, without any difference between the dissolution media, due to high solubility in both fasted and fed state fluids. In spite of the high dissolution rate of EV in both fasted and fed state, the poor permeability in the gastrointestinal tract of the hydrophilic compound caused lowered absorption in post-prandial condition [12]. Dissolution rates of the lipidic compound in biorelevant media were retarded compared to those of the parent compound (Figure 6). Nevertheless, in all biorelevant media, more than 50% of the EV-C12 added was released within 30 min and over 90% of the prodrug was dissolved within 2 h, regardless of pH, bile acid level, or salt concentration in the medium. The dissolution rate of EV-C12 in FeSSGF- and FeSSIF-simulating fed state were slightly higher than those in FaSSGF and FaSSIF, in accordance with solubility data presented earlier. Correspondingly, the release rate constants of EV-C12 powder in biorelevant media calculated using Hixon-Crowell cube-root model were 0.037 (*r^2^* = 0.866), 0.036 (*r^2^* = 0.914), 0.048 (*r^2^* = 0.879), and 0.053 (*r^2^* = 0.933) mg^1/3^/h. The high cumulative release of the lipophilic prodrug in all biorelevant media was expected to increase oral bioavailability of EV in both fasted and fed states, unlike EV within BCS class III, as the intestinal absorption of a lipophilic compound is highly dependent on the dissolution profile in aqueous media. Generally, BCS class II or IV compounds with high lipophilicity are more likely to show a positive food effect due to escalated in vivo solubility in the fed state [31]. Nevertheless, additional pharmaceutical approaches such as particle size reduction, solid dispersion and other solubilization techniques might be needed to further facilitate the dissolution rate and intestinal absorption of EV-C12. The concentration of EV in a dissolution medium formed by hydrolysis of this lipidic compound was found to be negligible (less than 1%) in all media. This result suggests that the ester compound could be stable in the gastrointestinal tract, preferably to be absorbed in an intact form in the stomach and/or small intestine.

## 3. Materials and Methods

### 3.1. Materials

All materials and reagents were obtained commercially. They were used without further purification unless otherwise specified. All the solvents utilized for analysis, routine product isolation, and chromatography were of analytical grade or reagent grade. Reaction flasks were dried at 100 °C before use. EV monohydrate powder with purity over 98.0 wt % was provided by Dong-A ST Pharmaceutical Co. (Seoul, Korea). Hexanoic acid, octanoic acid, decanoic acid, and dodecanoic acid, sodium taurocholate, lecithin, glyceryl monooleate, sodium oleate, acetic acid, sodium acetate, and sodium malate were obtained from Sigma Chemical Co. (St. Louis, MO, USA). Acetonitrile, isopropyl alcohol (IPA) and methanol of HPLC grade were purchased from J.T. Baker (Phillipsburg, NJ, USA). Flash column chromatography was performed using silica gel 60 (230–400 mesh, Merck, Billerica, MA, USA) with indicated solvents. Thin-layer chromatography (TLC) was performed using silica gel F_254_ plates (Merck). ^1^H and ^13^C spectra for characterization of EV-lipid conjugates were recorded on a Bruker advance III 800 (800 MHz) spectrometer in DMSO-*d_6_*. Chemical shifts are expressed in parts per million (ppm, δ) downfield from tetramethylsilane and referenced to a deuterated solvent. ^1^H NMR data are reported in the order of chemical shift, multiplicity (s, singlet; d, doublet; t, triplet; q, quartet; m, multiplet; and/or multiple resonances), number of protons, and coupling constant in hertz (Hz). Low-resolution mass spectra (LRMS) were obtained using an Advion Expression CMS and recorded in positive ion mode with an electrospray (ESI) source.

### 3.2. Synthesis of EV-FA Conjugates

#### 3.2.1. General Synthesis Procedure

To a solution of EV hydrate (500 mg, 1.69 mmol) in pyridine (20 mL), corresponding FA (1 equiv.), *N*,*N*-dimethylaminopyridine (DMAP, 0.05 equiv.), and 1-ethyl-3-(3-dimethylaminopropyl) carbodiimide (EDC, 2 equiv.) were added at ambient temperature. The reaction mixture was stirred for 6 h at the same temperature, quenched by water, concentrated in vacuo, and diluted with 10% methanol in dichloromethane and water. The organic layer was washed with 1N hydrochloric acid and brine, dried over magnesium sulfate, and concentrated in vacuo. Purification of the residue via flash column chromatography on silica gel (methanol/chloroform = 1:20~1:10) afforded the titled compound as a white solid.

#### 3.2.2. (1*R*,3*S*,5*S*)-3-(2-Amino-6-oxo-1*H*-purin-9(6*H*)-yl)-5-hydroxy-2-methylenecyclopentyl)methyl hexanoate (EV-C6)

Yield: 41%; ^1^H-NMR (800 MHz, DMSO-*d_6_*): δ 10.64 (s, 1H), 7.66 (s, 1H), 6.44 (s, 2H), 5.39–5.36 (m, 1H), 5.15–5.14 (m, 1H), 5.08 (s, 1H), 4.61 (s, 1H), 4.20–4.16 (m, 3H), 2.74–2.72 (m, 1H), 2.33 (t, 2H, *J* = 7.4 Hz), 2.29–2.27 (m, 1H), 2.08–2.06 (m, 1H), 1.54 (t, 2H, *J* = 7.3 Hz), 1.30–1.20 (m, 4H), 0.85 (t, 3H, *J* = 6.9 Hz); ^13^C-NMR (200 MHz, DMSO-*d_6_*): δ 173.0, 156.8, 153.5, 151.3, 150.0, 135.9, 116.4, 110.3, 70.2, 64.5, 54.9, 50.3, 38.6, 33.5, 30.6, 24.1, 21.8, 13.8; MS (ESI) *m*/*z*: 376 [M + H^+^].

#### 3.2.3. ((1*R*,3*S*,5*S*)-3-(2-Amino-6-oxo-1*H*-purin-9(6*H*)-yl)-5-hydroxy-2-methylenecyclopentyl)methyl octanoate (EV-C8)

Yield: 51%; ^1^H-NMR (800 MHz, DMSO-*d_6_*): δ 10.63 (s, 1H), 7.66 (s, 1H), 6.45 (s, 2H), 5.37 (td, 1H, *J* = 2.3, 7.9 Hz), 5.15 (t, 1H, *J* = 2.3 Hz), 5.08 (s, 1H), 4.61 (t, 1H, *J* = 2.2 Hz), 4.20–4.16 (m, 3H), 2.74–2.72 (m, 1H), 2.33–2.27 (m, 3H), 2.09–2.05 (m, 1H), 1.53 (t, 2H, *J* = 7.2 Hz), 1.25–1.22 (m, 8H), 0.85 (t, 3H, *J* = 6.9 Hz); ^13^C-NMR (200 MHz, DMSO-*d_6_*): δ 173.0, 156.8, 153.5, 151.3, 150.0, 135.9, 116.4, 110.3, 70.2, 64.5, 54.9, 50.3, 38.6, 33.5, 31.1, 28.4, 28.3, 24.5, 22.0, 13.9; MS (ESI) *m*/*z*: 404 [M + H^+^].

#### 3.2.4. ((1*R*,3*S*,5*S*)-3-(2-Amino-6-oxo-1*H*-purin-9(6*H*)-yl)-5-hydroxy-2-methylenecyclopentyl)methyl decanoate (EV-C10)

Yield: 49%; ^1^H-NMR (800 MHz, DMSO-*d_6_*): δ 10.61 (s, 1H), 7.66 (s, 1H), 6.42 (s, 2H), 5.38 (t, 1H, *J* = 8.0 Hz), 5.15 (t, 1H, *J* = 2.1 Hz), 5.08 (s, 1H), 4.62 (t, 1H, *J* = 2.2 Hz), 4.20–4.16 (m, 3H), 2.73–2.72 (m, 1H), 2.33–2.28 (m, 3H), 2.09–2.06 (m, 1H), 1.53 (t, 2H, *J* = 7.1 Hz), 1.26–1.22 (m, 12H), 0.84 (t, 3H, *J* = 6.9 Hz); ^13^C-NMR (200 MHz, DMSO-*d_6_*): δ 173.0, 156.8, 153.5, 151.3, 150.0, 135.9, 116.4, 110.3, 70.2, 64.5, 54.9, 50.3, 38.6, 33.5, 31.2, 28.8, 28.7, 28.6, 28.4, 24.5, 22.1, 13.9; MS (ESI) *m*/*z*: 432 [M + H^+^].

#### 3.2.5. ((1*R*,3*S*,5*S*)-3-(2-Amino-6-oxo-1*H*-purin-9(6*H*)-yl)-5-hydroxy-2-methylenecyclopentyl)methyl dodecanoate (EV-C12)

Yield: 47%; ^1^H-NMR (800 MHz, DMSO-*d_6_*): δ 10.64 (s, 1H), 7.66 (s, 1H), 6.46 (s, 2H), 5.38–5.36 (m, 1H), 5.14 (t, 1H, *J* = 2.2 Hz), 5.09 (s, 1H), 4.61 (t, 1H, *J* = 2.2 Hz), 4.20–4.15 (m, 3H), 2.73–2.72 (m, 1H), 2.33–2.28 (m, 3H), 2.08–2.06 (m, 1H), 1.54 (t, 2H, *J* = 7.3 Hz), 1.27–1.22 (m, 16H), 0.84 (t, 3H, *J* = 6.9 Hz); ^13^C-NMR (200 MHz, DMSO-*d_6_*): δ 173.0, 156.8, 153.5, 151.3, 150.0, 135.8, 116.4, 110.2, 70.2, 64.5, 54.9, 50.3, 38.6, 33.5, 31.3, 28.96, 28.95, 28.86, 28.67, 28.66, 28.42, 24.5, 22.1, 13.9; MS (ESI) *m*/*z*: 460 [M + H^+^].

### 3.3. HPLC Determination of EV-FA Conjugates

Concentrations of EV and their lipidic conjugates in samples were quantitated using an isocratic HPLC system. HPLC analysis conditions of EV and its derivatives are summarized in Table 2. The Waters HPLC system was comprised of a pump (Model 515 pump), an autosampler (Model 71P auto sampler), and a UV detector (Model 486 UV detector) equipped with a C18 column (150 mm × 4.6 mm, 3 μm). The mobile phase of EV consisted of a mixture of acetonitrile and distilled water at a ratio of 4:96 (pH 2.8, adjusted with trifluoroacetic acid). On the other hand, the mobile phase for the analysis of EV derivatives consisted of IPA and distilled water at a ratio of 30:70 to 50:50. The column temperature was set at 25 °C in a column oven. Eluent was monitored at a wavelength of 253 nm. All calibration curves in the concentration range of 1.0‒100 μg/mL were linear, with correlation coefficient (*r*^2^) values over 0.9995 (Table 2). 

### 3.4. Determination of Aqueous Solubility, logP, and Acidic Hydrolysis of EV-FAs

The equilibrium solubilities of lipidic prodrugs in biorelevant media such as FaSSGF, FeSSGF, FaSSIF, and FeSSIF [32,33] were assessed. FaSSGF was prepared with 0.08 mM of sodium taurocholate, 0.02 mM of lecithin, and 34.2 mM of NaCl in 1000 mL of distilled water (pH 1.6). FaSSIF was prepared with 3 mM of sodium taurocholate, 0.2 mM of lecithin, 19.1 mM of malate, 68.62 mM of NaCl, and 34.8 mM of NaOH in 1000 mL of distilled water (pH 6.5, osmolality: 270 mOsm∙kg^−1^). FeSSGF was prepared with 230.0 mM of NaCl, 17.1 mM of acetic acid, and 29.75 mM of sodium acetate, in 1000 mL of mixture of milk (full fat 3.5%) and distilled water (pH 5.0, osmolality: 400 mOsm∙kg^−1^). FeSSIF was prepared with 10 mM of sodium taurocholate, 2 mM of lecithin, 5 mM of glyceryl monooleate, 0.8 mM of sodium oleate, 55.0 mM of malate, 125.5 mM of NaCl, and 81.6 mM of NaOH in 1000 mL of distilled water (pH 5.8, osmolality: 390 mOsm∙kg^−1^).

An excess amount of drug (500 mg) was added to biorelevant media in a scintillation vial. Drug suspensions were sonicated for 30 min and then shaken at 37 °C for 3 days using a shaking incubator. Samples were then centrifuged at 13,000 rpm for 10 min and the supernatant was appropriately diluted with mobile phase. The concentration of EV-FA in the medium was analyzed using HPLC as described above.

Log*P* values of the EV derivatives defined as the concentration ratio of compounds between two immiscible media (octanol and distilled water) at equilibrated state were calculated using ChemDraw Ultra 7.0 (version 3.0, BioByte Corp., Claremont, CA, USA) [34].

To estimate the chemical stability of these synthesized ester prodrugs in the stomach, their hydrolysis behavior was evaluated in simulated gastric fluid (pH 1.2). Each compound (10 mg) was dissolved in ethanol (10 mL) and then serially diluted with simulated gastric fluid at a drug concentration of 20 μg/mL. After incubation at 37 °C for 24 h in a shaking incubator, samples were withdrawn and analyzed using HPLC, as described above.

### 3.5. Physical Characterization of EV-C12

Solid state morphology, thermal properties, and the X-ray diffraction patterns of EV-C12 (the optimized EV prodrug chosen based on aqueous stability, log*P*, and the degree of acidic hydrolysis) were assessed. The morphological features of EV-C12 powder was observed by scanning electron microscopy (SEM, Jeol JSM 6510 SEM, Akishima, Japan). The powder was fixed on a disc with double-sided adhesive carbon tape and coated with platinum by an automatic magnetron sputter coater system (Jeol MSC201, Akishima, Japan) in a vacuum for 4 min at 15 mA. Microphotographs were then taken at an acceleration voltage of 15 kV.

The thermal properties of the lipidic prodrug EV-C12 were then analyzed using a DSC (DSC 50, Shimadzu Scientific Instruments, Kyoto, Japan). About 2 mg of the sample was enclosed in a Tzero pan and lid and subjected to heating at a scanning rate of 10 °C/minute over the range of 35 to 285 °C under nitrogen gas purging. A flow rate of gas purging was set at 20 mL/minute.

Crystallinity of the powder was evaluated using an XRD (X’Pert Prompt PANalytical Co., Lelyweg, The Netherlands) at room temperature. A monochromatic Cu Kα-radiation (λ = 1.5418 Å) at current of 30 mA and voltage of 40 kV was applied to the powder. The diffraction pattern over 2θ range of 10 to 40° was determined using a step size of 0.02° at a scan speed of 1 s/step.

### 3.6. In Vitro Dissolution Test

The United States Pharmacopeia 29 paddle method was used to assess the release profile of EV-C12 powder using a VK 7000 dissolution tester (Varian Medical Industries, Palo Alto, CA, USA). The EV-FA powder equivalent to 0.5 mg of the parent compound was immersed in 900 mL of biorelevant media maintained at 37 °C ± 0.1 °C at a paddle speed of 50 rpm. At fixed time intervals, aliquots (about 4 mL) were drawn and centrifuged at 13,000 rpm for 10 min. An equal volume of the test medium pre-warmed at 37 °C ± 0.1 °C was used for immediate replacement. The supernatant was appropriately diluted with methanol and the drug concentration in the supernatant was assayed by HPLC, as described earlier.

### 3.7. Statistical Analysis

Statistical analysis was carried out using an analysis of variance (ANOVA) with Tukey’s HSD (honest significant difference) test or *t*-test, in order to compare the hydrolysis rate of lipidic prodrugs or the dissolution profile between EV and EV-C12. The *p* value below 0.05 was considered to be significant.

## 4. Conclusions

To increase intestinal absorption of EV, a BCS III compound, a novel lipidic prodrug was designed in this paper. Pure lipid conjugates of EV were prepared in moderate yields (41~51%) by selective esterification of the primary alcohol of EV with diverse FAs (C6~C12) using EDC, followed by purification via flash column chromatography on silica gel. EV-C12 with an acceptable Clog*P* value (3.62) and the lowest hydrolysis rate in simulated gastric fluid (22.5% for 12 h, pH 1.2) was chosen as a prodrug candidate. During in vitro release tests in various biorelevant media, more than 90% of the prodrug was released within 2 h in all dissolution media, simulating both fasted and fed states. Further investigations, including in vivo pharmacokinetic studies and preclinical tolerability tests, are needed to estimate the potential of this novel prodrug as an alternative for current EV therapy to alleviate food effect on intestinal absorption of EV.

## Figures and Tables

**Table 1:** Equilibrium solubility ^1^ in biorelevant media and Clog*P* values of EV and EV-FA conjugates.

	FaSSGF (μg/mL)	FaSSIF (μg/mL)	FeSSGF (μg/mL)	FeSSIF (μg/mL)	Clog*P* ^2^
EV	8940 ± 92	1624 ± 23	2152 ± 13	2241 ± 52	−2.03
EV-C6	4.84 ± 0.5	5.54 ± 0.6	24.9 ± 1.4	25.2 ± 2.3	0.45
EV-C8	4.74 ± 0.3	5.74 ± 0.3	38.2 ± 1.5	36.7 ± 1.2	1.51
EV-C10	4.41 ± 0.1	6.47 ± 0.6	61.9 ±3.2	66.6 ± 4.4	2.56
EV-C12	4.82 ± 0.2	6.50 ± 0.2	76.6 ± 4.0	78.8 ± 7.7	3.62

^1^ Solubility data are expressed as mean ± SD (*n* = 3); ^2^ Calculated using ChemDraw Ultra 7.0 program.

**Table 2:** HPLC analysis conditions for EV-lipid conjugates and calibration curve equations.

	Mobile Phase	Calibration Curve	Linearity (*r*^2^ Value)
EV	ACN:DW = 4:96 (pH 2.8) ^1^	y=23777x−226.4	1.0000
EV-C6	IPA:DW = 30:70	y=38223x−5956.6	0.9995
EV-C8	IPA:DW = 40:60	y=32403x+27852	0.9996
EV-C10	IPA:DW = 40:60	y=30973x−15578	0.9998
EV-C12	IPA:DW = 50:50	y=32292x−689.4	0.9998

^1^ pH of mobile phase of EV was set to 2.8, by adding 0.002% *v*/*v* of trifluoroacetic acid dropwise; Other HPLC analytical conditions except mobile phase composition were the same for each compounds: C18 column (150 mm × 4.6 mm, 3 μm), column temperature of 25 °C, flow rate of 1.0 mL/min, injection volume of 20 μL, and wavelength of 253 nm.
